# Field test of a novel detection device for *Mycobacterium tuberculosis *antigen in cough

**DOI:** 10.1186/1471-2334-10-161

**Published:** 2010-06-08

**Authors:** Ruth McNerney, Beyene A Wondafrash, Kebede Amena, Ato Tesfaye, Elaine M McCash, Nicol J Murray

**Affiliations:** 1 Department of Infectious and Tropical Diseases, London School of Hygiene & Tropical Medicine, Keppel Street, London, WC1E 7HT, UK; 2 Adama Hospital, PO BOX 611, Nazareth, Oromia 04, Ethiopia; 3 Rapid Biosensor Systems Ltd, Babraham Hall, Babraham, Cambridge, CB22 3AT, UK

## Abstract

**Background:**

Tuberculosis is a highly infectious disease that is spread from person to person by infected aerosols emitted by patients with respiratory forms of the disease. We describe a novel device that utilizes immunosensor and bio-optical technology to detect *M. tuberculosis *antigen (Ag85B) in cough and demonstrate its use under field conditions during a pilot study in an area of high TB incidence.

**Methods:**

The TB Breathalyzer device (Rapid Biosensor Systems Ltd) was field tested in the outpatient clinic of Adama Hospital, Ethiopia. Adults seeking diagnosis for respiratory complaints were tested. Following nebulization with 0.9% saline patients were asked to cough into a disposable collection device where cough aerosols were deposited. Devices were then inserted into a portable instrument to assess whether antigen was present in the sample. Demographic and clinical data were recorded and all patients were subjected to chest radiogram and examination of sputum by Ziehl-Nielsen microscopy. In the absence of culture treatment decisions were based on smear microscopy, chest x-ray and clinical assessment. Breathalyzer testing was undertaken by a separate physician to triage and diagnostic assessment.

**Results:**

Sixty individuals were each subjected to a breathalyzer test. The procedure was well tolerated and for each patient the testing was completed in less than 10 min. Positive breath test results were recorded for 29 (48%) patients. Of 31 patients with a diagnosis of tuberculosis 23 (74%; 95% CI 55-87) were found positive for antigen in their breath and 20 (64%; 95% CI 45-80) were smear positive for acid fast bacilli in their sputum. Six patients provided apparent false positive breathalyzer results that did not correlate with a diagnosis of tuberculosis.

**Conclusions:**

We propose that the breathalyzer device described warrants further investigation as a tool for studying exhalation of *M. tuberculosis*. The portability, simplicity of use and speed of the test device suggest it may also find use as a tool to aid early identification of infectious cases. We recommend studies be undertaken to determine the diagnostic sensitivity and specificity of the device when compared to microbiological and clinical indicators of tuberculosis disease.

## Background

With an estimated annual incidence of over nine million cases tuberculosis (TB) is believed to be responsible for more adult deaths each year than any other single infectious agent [1]. The highest burden of disease is currently borne by the less developed countries of Africa and Asia where efforts to control TB are hampered by weak health systems and in some settings, by the high prevalence of co-infection with HIV [2]. The recent emergence of multi drug-resistant stains that cannot be cured with standard treatment regimens has served to emphasize the urgency of the situation [3,4]. Control of TB in high burden countries relies on the detection and treatment of infectious cases, most usually by testing patients attending a health clinic that report a cough of at least three weeks duration. The diagnostic tests available in these settings are sputum smear microscopy, an insensitive technique requiring a skilled practitioner and chest radiography, a technique lacking in specificity as well as sensitivity [5]. World Health Organization estimates suggest that in 2006 there were 4 million individuals with undiagnosed tuberculosis. More effective interventions are required to detect and treat infectious cases earlier in the transmission chain, particularly in vulnerable communities with a high prevalence of HIV.

*Mycobacterium tuberculosis*, the causative agent is spread from person to person via infected aerosols created by patients with respiratory forms of the disease. Bacilli released into the airways following necrosis and destruction of lung tissue may be expelled from the lungs and if released in the form of aerosols may remain airborne and available for inhalation and infection of a new host. Despite being the major mode of transmission there is little data available regarding the exhalation of *M. tuberculosis*. Retrospective study of TB contacts suggests that most transmission within households occurs prior to diagnosis and the initiation of treatment [6]. Advanced cavitary disease and the presence of high numbers of *M. tuberculosis *in expectorated sputum is associated with transmission [7] but it is not known how early in the infection that patients pose a significant risk of infecting others. There is little data regarding the aerosolization of bacilli by individual patients and increased knowledge regarding shedding of bacteria through the course of a tuberculosis infection would greatly enhance our understanding of transmission dynamics and spread of the disease. Epidemiological studies suggest there is considerable variation in infectiousness, with some patients infecting a large number of contacts whereas others apparently fail to pass on the disease [8-10]. Previous attempts to assess airborne transmission have relied on exposure of guinea pigs to hospitalized TB patients [11,12]. After a period of living in contact with air from the ward animals were tuberculin tested, followed by autopsy and assessment of lesions within the lungs. An alternative was recently offered by Fennelly and colleagues who developed a device for capturing cough generated aerosols from individual patients [13]. Samples of 'cough' were subjected to culture to assess the numbers of colony forming units of *M. tuberculosis*. Results were obtained in 6 to 10 weeks and showed variation in the quantities of culturable aerosols produced by individual patients [13]. Detection of specific nucleic acids following PCR amplification may provide an alternative means of detecting *M. tuberculosis *in aerosols where the DNA can be extracted from filters used to trap the particles and results obtained the next day [14]. When applied to air sampled in a hospital in Taiwan this methodology proved very sensitive as positive samples were obtained not only in the vicinity of known tuberculosis patients but in all parts of the hospital that were tested [15]. Rapid point-of-care methods of assessing whether an individual is expelling *M. tuberculosis *in their breath have not previously been reported. Such a device would greatly facilitate investigation of individual patients and so enable greater understanding of the factors influencing TB transmission.

We describe here a novel device that utilizes immunosensor and bio-optical technology to detect *M. tuberculosis *antigen in the breath of human subjects. The RBS Breathalyzer system (Rapid Biosensor Systems Ltd, Cambridge, UK) is a fully portable device comprising a disposable plastic collection tube (3.5 × 10 cm) into which the patient coughs [16]. The collection tube has been designed to collect aerosols and particles coughed out by the patient. After coughing an internal concentric plunger is suppressed to collect sample from the sides of the tube. A twisting action then distributes sample across the surface of a coated prism at the base of the tube where the detection reaction takes place. The prism is coated with anti-tuberculosis antibodies that are primed with analogue molecules labeled with a fluorescent dye. The analogue comprises artificially modified peptide subsequences of the T-cell epitope from *M. tuberculosis *Ag85B [17]. When native *M. tuberculosis *Ag85B antigens are presented they are able to displace the analogue, causing a change in the fluorescent signal. Changes in fluorescence are monitored by inserting the collection tube into a small battery powered instrument containing a diode laser and a photo-multiplier tube. The device utilises evanescent wave fluorimetry to assess the amount of antigen present at the surface of the prism [18]. A digital readout is provided within minutes and the whole test, including sample collection may be performed in less than 10 minutes.

Following a period of assessment by the test developers (Rapid Biosensor Systems Ltd) an independent study was performed in a country with a high prevalence of tuberculosis to assess whether the device was able detect TB antigen in cough of patients when used in the field. For this pilot study a prototype instrument was transported to Ethiopia where it was applied in the outpatient clinic of Adama Hospital, a referral hospital in Nazareth town, in the central region of Oromia.

## Results

A single breathalyzer test was performed on 60 consenting adults presenting with respiratory problems. Samples for analysis were collected in disposable collection tubes. Each individual was shown how to cough into the tube and requested not to attempt to expectorate sputum. Each patent coughed at least three times into the device (short coughs in rapid succession). The procedure was well tolerated by all participants and the total time taken for sample collection did not exceed 5 minutes. Sample collection tubes consisted of two concentric plastic components. Once samples had been collected the inner plunger was depressed and rotated to distribute the sample on the prism test surface. Tubes were then placed in the instrument. The initial digital readout was recorded and the reading observed over a period of two minutes. Samples that induced a reduction in signal greater than 20 units within two minutes were classified as positive for *M. tuberculosis *antigen; those that did not induce a reduction of 20 units were classified as negative for *M. tuberculosis *antigen. The manufacturer's protocol instructed that an increase in signal greater than 40 units be taken to indicate an invalid test; however, such readings were not observed during this study. Patients were classified according to their clinical presentation, chest radiography and sputum microscopy.

The mean age was 33.9 yrs (range 17-70) and ratio of male to female participants 29:31. There was no medical complaint other than respiratory illness reported by the patients during the triage; however, one individual was a known diabetic. All individuals tolerated the breathalyzer procedure well and a result was obtained for each patient tested. The time required for the combined nebulization, sample collection and breathalyzer test did not exceed 10 min, including the time taken to instruct the patient. Positive breath test results were recorded for 29 (48.3%) patients. Of 31 patients with a final diagnosis of TB 20 (65%; 95% CI 45-80) were found positive for acid fast bacilli in their sputum and 23 (74%; 95% CI 55-87)) were positive for antigen in their breath. When the breath antigen test result was combined with sputum microscopy 29/31 (94%; 95% CI 77-99) of clinically diagnosed TB patients were detected. The distribution of breathalyzer results is shown in Table 1. Nine smear negative patients with a clinical diagnosis of tuberculosis were positive for antigen in their breath. Six patients provided positive breathalyzer results that did not correlate with a diagnosis of tuberculosis suggesting a specificity of 79% (95% CI 60-91). As shown in Table 2, all six were found negative for acid fast bacilli in their sputum. Six patients positive for acid fast bacilli in their sputum were found negative in the breathalyzer test. A further two patients negative in both the breath test and by smear microscopy were given a final diagnosis of tuberculosis, one of whom (P30) admitted during further investigation that he had previously received treatment for pulmonary tuberculosis and was classified as an immunocompromised re-treatment case. The remaining 23 patients found negative for TB antigen were diagnosed as having pneumonia (6), bronchitis (5), upper respiratory tract infection (5), COPD (4), congestive heart failure (1), aseptic pneumonia (1) and echinococcal liver cyst (1).

**Table 1:** Results of Breathalyzer test for *M. tuberculosis *antigen 85B

Diagnosis	Breathalyzer test positive	Breathalyzer test negative	Total
**Smear positive TB**	14	6	20
**Smear negative TB**	9	2	11
**Not TB**	6	23	29
**Total**	29	31	60

**Table 2:** False positive and false negative Breathalyzer test results.

Patient	Breath test	Cough	Sputum microscopy	CXR report	Diagnosis
**P9**	Positive	Bloody	Negative	Bronchoectasis	Pneumonia
**P29**	Positive	Productive	Negative	Pneumonia	Pneumonia
**P19**	Positive	Dry	Negative	No finding	Bronchitis
**P31**	Positive	Productive	Negative	No finding	Bronchitis
**P32**	Positive	Purulent	Negative	Bronchoectasis	COPD
**P49**	Positive	Scanty	Negative	No finding	URTI
**P10**	Negative	Productive	Negative	Pneumonia	Possible PTB
**P22**	Negative	Productive	AFB	PTB	PTB
**P29**	Negative	Purulent	AFB	PTB	PTB
**P30**	Negative	Scanty	Negative	No finding	Re-treatment PTB
**P37**	Negative	Dry	AFB	No finding	PTB
**P39**	Negative	Bloody	AFB	Pleural effusion	PTB
**P42**	Negative	Dry	AFB	No finding	PTB
**P53**	Negative	Bloody	AFB	Disseminated TB	PTB

## Discussion

The factors influencing transmission of tuberculosis amongst individual patients are poorly understood. Improved understanding of the shedding of *Mycobacterium tuberculosis *by individual patients would greatly facilitate efforts to control the disease. We have demonstrated a novel portable device for assessing TB antigen in breath. This is the first report of a device that permits 'real time' monitoring of exhaled *M. tuberculosis*. It is also the first report of a 'point of care' test that would allow assessment of individuals in the community whilst they undertake their normal daily routines. Having demonstrated that the test can be performed in a clinic with minimal training further study is now required to determine the sensitivity of the device when compared to other markers of infection such as the number of bacilli in expectorated sputum. At the time of this study smear microscopy readings in Ethiopia were not graded according to the numbers of bacilli observed and further studies should be undertaken to assess the relationship between depression of signal in the breathalyzer test and smear score. Isolation and culture of the bacteria from expectorated sputum remains the most sensitive diagnostic tool for pulmonary tuberculosis and should necessarily be included in such studies. The viability of bacteria present in breath samples should also be ascertained and their identity confirmed by molecular testing to determine the specificity of the test. A limitation of this study was that each patient was subject to a single test. Studies on expectorated sputum suggest there is considerable variation in the number of bacilli present in sequentially collected sputum and similar variations in the amount of antigen in cough may occur. To investigate this phenomenon the breathalyzer test should ideally be performed on multiple occasions throughout the day. It should also be noted that the breathalyzer device does not discriminate the size of the particles detected. Studies of TB transmission indicate that the size of the infected aerosol or particle is critical to its ability to reach and infiltrate the lung [19,20]. Whereas smaller particles (less than one micron) are not stable and are quickly dispersed, large droplets of greater than 5 micron, should they be inhaled are less likely to reach the alveoli.

The breathalyzer device measures displacement of the analogue by antigen in the sample and the larger the number of antigens the greater the depression of signal. However, the absolute signal that the machine displays is a complex function involving a range of different optical and biological parameters and the relationship of antigen concentration to the final readout should be considered semi quantitative only. The signal displayed is dependant on both the fluorescent signal from the coating on the prism and also optical scattering effects. A decrease of less than 20 units is classed as negative which takes into account scattering changes if samples 'settle' in position or evaporation takes place. The cut off value was attained from unblinded clinical studies undertaken by the test inventors. The limit of sensitivity of the prototype device used in this study was not determined but is anticipated to be in the range of 50-75 colony forming units. Further studies are required to determine the sensitivity and specificity of the prism based displacement assay used in this device, these should include testing other mycobacteria species that may share the Antigen 85 epitopes on which the displacement assay has been based.

The failure to provide data by the Ethiopian laboratory contracted to undertake culture of the specimens demonstrates the fragility of laboratory based diagnostics in Ethiopia and illustrates the need for simple 'point of care devices' in such settings. Considerable laboratory infrastructure is required to undertake culture of *M. tuberculosis *in a controlled and safe environment and such facilities are rare in Africa. It should also be noted that although culture would be expected to provide a more sensitive means of diagnosis the method is slow and results obtained after a delay of several weeks would be of limited value in this setting. In this study six smear positive, presumed TB patients were found negative by the breathalyzer test. Thus the test may not be considered a replacement diagnostic test for smear microscopy. However, when positive data from the two tests were combined 93.5% of patients were identified suggesting the device might have a role in assisting diagnosis, particularly in those patients unable to expectorate sputum such as children or the severely immunocompromised. That TB antigen was not observed in the breath of some smear positive patients may seem surprising. However, some evidence to support the finding that bacteria are not constantly exhaled by patients has been provided by Fennelly and colleagues who, when measuring cultivable cough-generated aerosols observed that some untreated smear positive TB cases were negative in their test [13]. That six patients provided apparent false positive breathalyzer results that did not correlate with a diagnosis of tuberculosis indicates a specificity in the region of 79%. Further investigation is required to determine the significance of such results using more sensitive diagnostic technologies such as culture and nucleic acid amplification tests. An important consideration for new technology is the cost of implementation. It is anticipated that should the disposable TB breathalyzer device be produced on a large scale it would become available for less than USD$5 and that labor costs would remain modest due to the rapid nature of the test. The reader is a low cost, portable, multi-use device that runs on rechargeable batteries and so its cost can be amortized over several years. Assessment of the potential benefits of this new technology requires further study to determine its capabilities either as a tool to screen individuals for exhalation of *M. tuberculosis *or as device to aid diagnosis of pulmonary tuberculosis.

## Conclusions

The Breathalyzer test shows considerable promise as a tool to investigate shedding of *M. tuberculosis *in cough. Further study is required to determine the sensitivity and specificity of the test and the relationship of excreted Ag85B with smear positivity. These preliminary findings suggest that although this novel testing device cannot replace sputum microscopy it might assist in the early identification of TB cases that cannot expectorate sputum for examination. Further study should be undertaken to assess its value in populations with restricted access to healthcare and where screening for early tuberculosis disease using conventional tools is not currently possible.

## Methods

The study was approved by the Research Ethics Committee of the London School of Hygiene & Tropical Medicine and the Medical Directorate of Adama Hospital. All participants provided prior written consent. Patients were recruited from the outpatients' clinic of Adama Hospital. The clinic operates a triage system, only those patents presenting with respiratory complaints were considered eligible for the study. The triage and breathalyzer test were performed by separate physicians and results were not shared to prior to completion of the diagnostic process. Demographic and clinical data were recorded to include sex, age, smoking habits, weight, body temperature, blood pressure, respiratory rate and description of cough. All patients were subjected to chest radiogram and examination of sputum by Ziehl-Nielsen microscopy. Samples were taken to assess haemoglobin, erythrocyte sedimentation rate and white blood cell counts. Prior to testing with the breathalyzer device patients used a nebuliser with 0.9% saline to lubricate the trachea using a hand operated pump (Easy Air Nebuliser Pump, Cameron Price, Birmingham, UK). To reduce risk of nosocomial transmission samples were collected in isolation in a ventilated room with the supervising physician using a personal respirator. Sample collection tubes were single use and were disposed of following their removal from the instrument. The breathalyzer test result was not used in the assessment or management of patients. Facilities to culture *M. tuberculosis *were not available at the study site. Sputum specimens were sent to another laboratory for investigation by culture on Lowenstein Jensen but no results were received.

## Competing interests

RM, BW, KA and AT have no financial interests and no conflicting interests. EMM and NJM have financial interest in Rapid Biosensor Systems Ltd who are assignees of patents 'Biological Measurement System' (PCT WO 02/084266 A2) and 'Bioassay and Peptides for use therein' (WO2007/072063) and developers of the test device described.

## Authors' contributions

RM instigated, designed and supervised the study, interpreted results and drafted the article. BAW designed the study, undertook the breathalyzer testing and data analysis. KA assisted with establishing and managing the study in Ethiopia. AT undertook triage and clinical investigation of study subjects. EMM and NJM provided technical knowledge, protocols and training for the breathalyzer test. All authors read and approved the final manuscript.

## Pre-publication history

The pre-publication history for this paper can be accessed here:

http://www.biomedcentral.com/1471-2334/10/161/prepub

## References

[B1] World Health Organisation Global tuberculosis control: surveillance, planning, financing 2009 Geneva: WHO

[B2] Corbett EL Marston B Churchyard GJ De Cock KM Tuberculosis in sub-Saharan Africa: opportunities, challenges, and change in the era of antiretroviral treatment Lancet 2006 367 9514 926 937 10.1016/S0140-6736(06)68383-9 16546541

[B3] Zager EM McNerney R Multidrug-resistant tuberculosis BMC Infect Dis 2008 8 10 10.1186/1471-2334-8-10 18221534 PMC2241599

[B4] Zignol M Hosseini MS Wright A Weezenbeek CL Nunn P Watt CJ Williams BG Dye C Global incidence of multidrug-resistant tuberculosis J Infect Dis 2006 194 4 479 485 10.1086/505877 16845631

[B5] Perkins MD Cunningham J Facing the crisis: improving the diagnosis of tuberculosis in the HIV era J Infect Dis 2007 196 Suppl 1 S15 27 10.1086/518656 17624822

[B6] Kamat SR Dawson JJ Devadatta S Fox W Janardhanam B Radhakrishna S Ramakrishnan CV Somasundaram PR Stott H Velu S A controlled study of the influence of segregation of tuberculous patients for one year on the attack rate of tuberculosis in a 5-year period in close family contacts in South India Bull World Health Organ 1966 34 4 517 532 5296379 PMC2475993

[B7] Marks SM Taylor Z Qualls NL Shrestha-Kuwahara RJ Wilce MA Nguyen CH Outcomes of contact investigations of infectious tuberculosis patients Am J Respir Crit Care Med 2000 162 6 2033 2038 11112109 10.1164/ajrccm.162.6.2004022 PMC5448278

[B8] Kato-Maeda M Small PM How molecular epidemiology has changed what we know about tuberculosis West J Med 2000 172 4 256 259 10.1136/ewjm.172.4.256 10778380 PMC1070841

[B9] Valway SE Sanchez MP Shinnick TF Orme I Agerton T Hoy D Jones JS Westmoreland H Onorato IM An outbreak involving extensive transmission of a virulent strain of Mycobacterium tuberculosis N Engl J Med 1998 338 10 633 639 10.1056/NEJM199803053381001 9486991

[B10] Fennelly KP Variability of airborne transmission of Mycobacterium tuberculosis: implications for control of tuberculosis in the HIV era Clin Infect Dis 2007 44 10 1358 1360 10.1086/516617 17443475

[B11] Riley RL Mills CC Nyka W Weinstock N Storey PB Sultan LU Riley MC Wells WF Aerial dissemination of pulmonary tuberculosis. A two-year study of contagion in a tuberculosis ward. 1959 Am J Epidemiol 1995 142 1 3 14 7785671 10.1093/oxfordjournals.aje.a117542

[B12] Escombe AR Oeser C Gilman RH Navincopa M Ticona E Martinez C Caviedes L Sheen P Gonzalez A Noakes C The detection of airborne transmission of tuberculosis from HIV-infected patients, using an in vivo air sampling model Clin Infect Dis 2007 44 10 1349 1357 10.1086/515397 17443474 PMC2912511

[B13] Fennelly KP Martyny JW Fulton KE Orme IM Cave DM Heifets LB Cough-generated aerosols of Mycobacterium tuberculosis: a new method to study infectiousness Am J Respir Crit Care Med 2004 169 5 604 609 10.1164/rccm.200308-1101OC 14656754

[B14] Schafer MP Fernback JE Jensen PA Sampling and analytical method development for qualitative assessment of airborne mycobacterial species of the Mycobacterium tuberculosis complex Am Ind Hyg Assoc J 1998 59 8 540 546 9725932 10.1080/15428119891010712

[B15] Chen PS Li CS Concentration profiles of airborne Mycobacterium tuberculosis in a hospital Aerosol Sci Tech 2008 42 3 194 200 10.1080/02786820801922953

[B16] McCash E Wheeler G Colby E Storkey M Stewart J Murray N Glauser A Biological measurement system 2008 Office UPaT. US: Rapid Biosensor Systems Limited

[B17] Mustafa AS Shaban FA Abal AT Al-Attiyah R Wiker HG Lundin KE Oftung F Huygen K Identification and HLA restriction of naturally derived Th1-cell epitopes from the secreted Mycobacterium tuberculosis antigen 85B recognized by antigen-specific human CD4(+) T-cell lines Infect Immun 2000 68 7 3933 3940 10.1128/IAI.68.7.3933-3940.2000 10858206 PMC101670

[B18] Banwell CN McCash EM Fundamentals of Molecular Spectroscopy 1994 4 Mcgraw-Hill Education - Europe (United States)

[B19] Wells WF On Air-Borne Infection Study II. Droplets and droplet nuclei American Journal of Hygiene 1934 20 611 618

[B20] Hatch TF Distribution and deposition of inhaled particles in respiratory tract Bacteriol Rev 1961 25 237 240 13905321 10.1128/br.25.3.237-240.1961 PMC441098

